# Anti-citrullinated peptide antibodies are the strongest predictor of clinically relevant radiographic progression in rheumatoid arthritis patients achieving remission or low disease activity: A post hoc analysis of a nationwide cohort in Japan

**DOI:** 10.1371/journal.pone.0175281

**Published:** 2017-05-15

**Authors:** Tomohiro Koga, Akitomo Okada, Takaaki Fukuda, Toshihiko Hidaka, Tomonori Ishii, Yukitaka Ueki, Takao Kodera, Munetoshi Nakashima, Yuichi Takahashi, Seiyo Honda, Yoshiro Horai, Ryu Watanabe, Hiroshi Okuno, Toshiyuki Aramaki, Tomomasa Izumiyama, Osamu Takai, Taiichiro Miyashita, Shuntaro Sato, Shin-ya Kawashiri, Naoki Iwamoto, Kunihiro Ichinose, Mami Tamai, Tomoki Origuchi, Hideki Nakamura, Kiyoshi Aoyagi, Katsumi Eguchi, Atsushi Kawakami, the Japanese RA Patients with RRP Study Group

**Affiliations:** 1 Center for Bioinformatics and Molecular Medicine, Nagasaki University Graduate School of Biomedical Sciences, Sakamoto 1-7-1, Nagasaki, Japan; 2 Unit of Translational Medicine, Department of Immunology and Rheumatology, Nagasaki University Graduate School of Biomedical Sciences, Sakamoto 1-7-1, Nagasaki, Japan; 3 Japanese Red Cross Nagasaki Genbaku Hospital, Department of Rheumatology, Mori-machi 3–15, Nagasaki, Japan; 4 Kurume University Medical Center, Department of Rheumatology, Kokubun 155–1, Kurume, Japan; 5 Zenjinkai Shimin-no-Mori Hospital, Shioji 2783–37, Miyazaki, Japan; 6 Tohoku University Hospital, Department of Hematology and Rheumatology, Aoba-ku, seiryo 1–1, Sendai, Japan; 7 Sasebo Chuo Hospital, Rheumatic and Collagen Disease Center, Yamato 15, Sasebo, Japan; 8 Tohoku Medical and Pharmaceutical University Hospital, Aoba-ku, komatsujima 4-4-1, Sendai, Japan; 9 Yu Family Clinic, Rifu-aza sinkan 2–5, Miyagi, Japan; 10 Kurume University School of Medicine, asahi-machi 67, Kurume, Japan; 11 Tohoku University Hospital, Department of Orthopaedic Surgery, Hirase 9–3, Sasebo, Japan; 12 East Sendai Rheumatism and internal medicine Clinic, Miyagino, Nittahigashi 1-17-5, Sendai, Japan; 13 Osaki Citizen Hospital, Furukawa-honami 3-8-1, Osaki, Japan; 14 NHO Nagasaki Medical Center, Kubara 2-1001-1, Omura, Nagasaki Japan; 15 Clinical Research Center, Nagasaki University Hospital, Sakamoto 1-7-1, Nagasaki, Japan; 16 Department of Public Health, Nagasaki University Graduate School of Biomedical Sciences, Sakamoto 1-12-4, Nagasaki, Japan; 17 Department of Rehabilitation Sciences, Nagasaki University Graduate School of Biomedical Sciences, Sakamoto 1-12-4, Nagasaki, Japan; 18 Sasebo City General Hospital, Hirase 9–3, Sasebo, Japan; Keio University, JAPAN

## Abstract

**Objectives:**

To determine prognostic factors of clinically relevant radiographic progression (CRRP) in patients with rheumatoid arthritis (RA) achieving remission or low disease activity (LDA) in clinical practice.

**Methods:**

Using data from a nationwide, multicenter, prospective study in Japan, we evaluated 198 biological disease-modifying antirheumatic drug (bDMARD)-naïve RA patients who were in remission or had LDA at study entry after being treated with conventional synthetic DMARDs (csDMARDs). CRRP was defined as the yearly progression of modified total Sharp score (mTSS) >3.0 U. We performed a multiple logistic regression analysis to explore the factors to predict CRRP at 1 year. We used receiver operating characteristic (ROC) curve to estimate the performance of relevant variables for predicting CRRP.

**Results:**

The mean Disease Activity Score in 28 joints-erythrocyte sedimentation rate (DAS28-ESR) was 2.32 ± 0.58 at study entry. During the 1-year observation, remission or LDA persisted in 72% of the patients. CRRP was observed in 7.6% of the patients. The multiple logistic regression analysis revealed that the independent variables to predict the development of CRRP were: anti-citrullinated peptide antibodies (ACPA) positivity at baseline (OR = 15.2, 95%CI 2.64–299), time-integrated DAS28-ESR during the 1 year post-baseline (7.85-unit increase, OR = 1.83, 95%CI 1.03–3.45), and the mTSS at baseline (13-unit increase, OR = 1.22, 95%CI 1.06–1.42).

**Conclusions:**

ACPA positivity was the strongest independent predictor of CRRP in patients with RA in remission or LDA. Physicians should recognize ACPA as a poor-prognosis factor regarding the radiographic outcome of RA, even among patients showing a clinically favorable response to DMARDs.

## Introduction

Rheumatoid arthritis (RA) is a systemic inflammatory disease characterized by autoimmune disorder and progressive joint destruction, leading to impaired quality of life [1, 2]. The therapeutic strategies for RA have developed remarkably, and the treat-to-target (T2T) strategy described in the European League Against Rheumatism (EULAR) recommendations is to aim for remission or low disease activity (LDA) [3]. However, some RA patients develop clinically relevant radiographic progression (CRRP) despite the achievement of remission or LDA by the T2T strategy with disease-modifying antirheumatic drug (DMARDs) [4].

We suspect that the development of CRRP is due to subclinical/residual synovitis. A number of previous studies attempted to identify promising prognostic markers of CRRP. Various clinical and biological markers including C-reactive protein (CRP) at baseline, erosion score at baseline, and the presence of autoantibodies have been identified as risk factors for CRRP in RA patients with high disease activity [5–8]. Importantly, the achievement of clinical remission defined by, for example, the Disease Activity Score in 28 joints (DAS28), the Simplified Disease Activity Index (SDAI) and the Clinical Disease Activity Index (CDAI) is not always associated with good structural and functional outcomes [9, 10]. In addition, subclinical synovitis or residual synovitis is well recognized in patients with RA and has been demonstrated by ultrasonography (US) [11–13] and MRI [14].

A Japanese Institute of Rheumatoid Arthritis (IORRA) observational study showed that more than one-half of real-world RA patients achieved clinical remission or LDA [15]. In daily practice it would be very useful to have prognostic markers for CRRP in 'good responders' among RA patients. However, little is known about variables that could be used to predict CRRP among RA patients who achieve clinical remission or LDA with DMARDs, especially conventional synthetic DMARDs (csDMARDs).

In the present study, using data from a nationwide, multicenter, prospective study in Japan, we evaluated a large number of clinical variables for their ability to predict the development of joint damage as CRRP after 1 year in RA patients who achieved remission or LDA with csDMARDs.

## Methods

### Patients

We performed a secondary analysis using data from a prospective, observational cohort study registered with the University Hospital Medical Information Network Clinical Trials Registry (UMIN-CTR) [http://www.umin.ac.jp/ctr/] (#UMIN000014791), conducted in daily clinical practices for RA in Japan. Overall, 887 RA patients from 26 centers affiliated with Nagasaki University or Tohoku University in Japan were recruited as the study cohort between May 2009 and March 2012. All of the patients were examined and treated by Japan College of *Rheumatology* (JCR)-certified rheumatologists.

Using this observational cohort, we recently reported prognostic factors for CRRP in RA patients whose clinical disease activity was moderate to high at enrollment [16]. In this second investigation, we focused on the prognostic factors for CRRP in 198 RA patients who achieved remission or LDA at enrollment.

When a patient relapsed during the present study, one of the participating JCR-certified rheumatologists treated the patient by using a T2T strategy that included the use of bDMARDs. We observed all of the patients for 1 year after their respective enrollment and assessed their RA disease activity every 3 months, using the DAS28-ESR and the Health Assessment Questionnaire (HAQ) [17]. The cumulative inflammatory burden was estimated by the time-integrated values (area under the curve-AUC) of DAS28-ESR. We constructed plots of longitudinal data of DAS28-ESR (baseline, 3 month, 6 month, 9 month and 12 month after enrollment) and calculated the area under the curve as described previously [18, 19].

All patients gave their signed informed consent to be subjected to the protocol, which was approved by the Institutional Review Board of Nagasaki University (approval number: 10022570–2), Tohoku University and the related centers.

### Structural damage assessment

To evaluated the patients' structural damage, radiographs of each patient's hands and feet were obtained at baseline and at 1 year, and the images were evaluated by two independent rheumatologists blinded to the clinical evaluation, using the van der Heijde-mTSS system as described [16, 20]. In our 2016 study [16], the interobserver reliability (as determined by the interclass correlation coefficient) was 0.97, and the smallest detectable change of mTSS in the present study was calculated as 2.96 as described [21]. Accordingly, we defined an annual increase of the mTSS >3.0 units as the development of CRRP, as was done in a previous study [22].

### Statistical analysis

The baseline demographic, clinical, and radiographic characteristics of the RA patients with and without CRRP were compared with Fisher's exact test for discrete variables, and with Wilcoxon’s test for continuous variables. To determine the independent predictive factors of the development of CRRP at 1 year, we performed a multiple logistic regression analysis. We selected variables with p-values <0.05 by univariate analyses as model 1. We then determined the final model by selecting the variables with p-values <0.05 in model 1. We also performed subgroup analysis based on disease activity at enrollment (remission vs. LDA). Each subgroup was analyzed with a multiple logistic regression analysis by selecting variables we used the final model. To convert continuous variables to binary variables, we defined the cut-off values by constructing a receiver operator characteristic (ROC) curve. Since treatment is an important confounder for radiographic progression, we performed a sensitivity analysis by removing patients treated with bDMARDs. Separation occurs in subgroup analysis because a contingency table of CRRP and ACPA had a zero cell. Separation leads diverges to infinity of estimated parameters. To solve this problem, we performed a Firth’s bias-reduced penalized-likelihood logistic regression [23]. This method is a modified logistic regression which can obtain estimates which do not diverge even in separation. The statistical analyses were performed using SAS 9.4 software (SAS Institute, Cary, NC, USA). A p-value <0.05 was considered significant.

## Results

### Patients

We analyzed 198 patients with remission or LDA at enrollment in this study. The patients’ characteristics are shown in Table 1. Their mean age was 58.8 years, and the median disease duration was 5.1 years. The mean DAS28-ESR at baseline was 2.33. Among the 198 patients, the treatment of 67 patients (34%) was strengthened with one or more csDMARDs or bDMARDs according to the EULAR recommendation, as decided by the participating rheumatologists. For eight patients, the bDMARDs (adalimumab [ADA], n = 1; etanercept [ETA], n = 2; infliximab [IFX], n = 2; tocilizumab [TCZ], n = 1; and abatacept [ABT], n = 2) were initiated within 3 months after the enrollment. In contrast, there were five patients (3%) whose treatment was weakened during the 1 year.

**Table 1:** Association between baseline characteristics and CRRP (univariate analyses)*.

Variables	All patients (n = 198)	CRRP (+) (n = 15)	CRRP(−) (n = 183)	p-value
Demographic:				
Age, yrs	58.8 (12.1)	59.0 (12.1)	55.9 (12.1)	0.22
Female, n (%)	144 (73)	14 (93)	130 (71)	0.073
Disease characteristics:				
Disease duration, years	5.1 (2.5–8.4)	4.5 (2.3–8.4)	5.2 (2.6–8.4)	0.98
RF positive, n (%)	140 (71)	13 (87)	127 (69)	0.24
ACPA positive, n (%)	113 (57)	14 (93)	99 (54)	0.0025
RF titer, U/ml	58 (17.5–122)	44 (26–626)	58 (17.3–122)	0.81
ACPA titer, U/ml	68.65 (13.6–100)	53.7 (48.8–75.9)	70.2 (12.5–102.5)	0.77
Disease activity:				
DAS28-ESR at baseline	2.33 (0.58)	2.49 (0.56)	2.31 (0.57)	0.27
Time-integrated DAS28-ESR	28.1 (23.0–33.7)	33.0 (24.4–41.2)	27.8 (23.0–33.5)	0.049
Relapse during the follow up	56 (28)	8 (53)	48 (26)	0.036
CRP at baseline, mg/dl	0.15 (0.07–0.36)	0.28 (0.13–0.92)	0.14 (0.07–0.32)	0.0093
ESR at baseline, mm/h	12 (8–19)	15 (8–25)	12 (8–18)	0.34
HAQ at baseline	0.16 (0.32)	0.25 (0.32)	0.15 (0.31)	0.037
Radiographs:				
mTSS at baseline	13 (5.5–35)	28 (10.5–104.5)	12.5 (5–32)	0.016
Erosion score at baseline	8 (3.5–21.5)	20 (7.5–66.5)	7.5 (3.5–20)	0.026
JSN score at baseline	5 (1–15.5)	16 (5–27)	5 (1–21)	0.0026
Treatment:				
Methotrexate use, n (%)	155 (78)	10 (67)	145 (80)	0.32
Dose of Methotrexate at baseline, mg/week	6.9 (1.9)	7.4 (2.1)	6.9 (1.9)	0.48
Maximum dose of Methotrexate during 1 year, mg/week	7.7 (2.1)	8.6 (1.6)	7.6 (2.1)	0.1
Prednisolone use, n (%)	76 (38)	3 (20)	73 (39)	0.17
bDMARDs introduction	8 (4)	1 (7)	7 (4)	0.47

*Mean values (SD), median (interquartile range) or number (percentages) are shown.

P-values were established using Fisher’s exact test or the Mann-Whitney U-test. CRRP: clinically relevant radiographic progression; RF: rheumatoid factor; ACPA: anti-citrullinated peptide antibodies; DAS28-ESR: Disease Activity Score 28 joints-erythrocyte sedimentation rate; CRP: C-reactive protein; HAQ: Health Assessment Questionnaire; mTSS: modified total Sharp score; JSN: joint space narrowing; bDMARD: biological disease-modifying antirheumatic drug.

**Fig 1:**
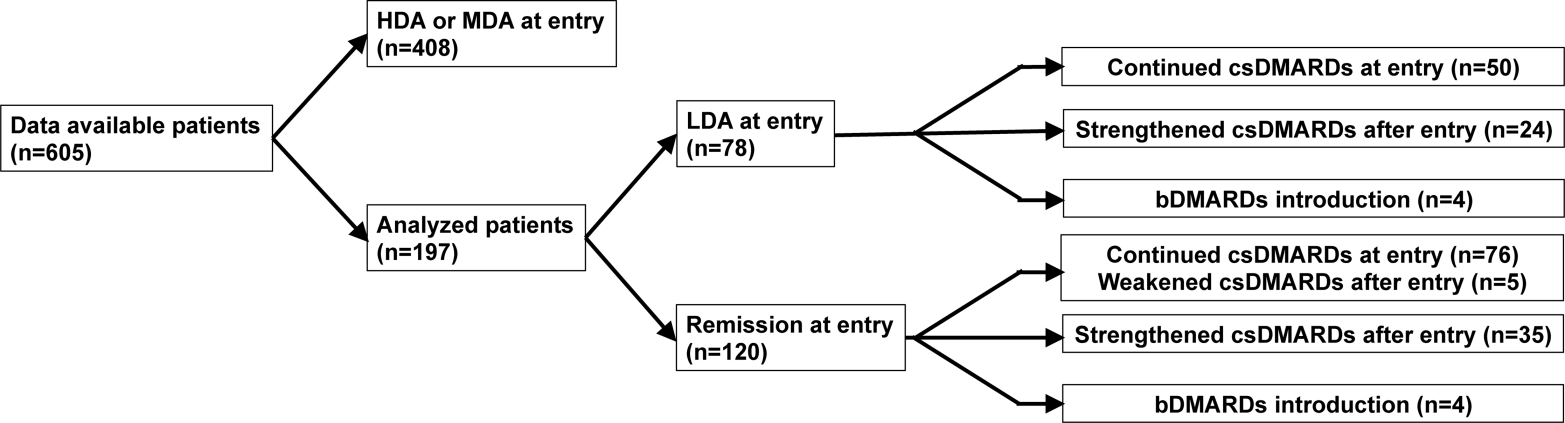
Patient enrollment flow chart and the therapeutic course during the 1-year observation after the baseline in our RA cohort. HDA: high disease activity; MDA: moderate disease activity; LDA: low disease activity; csDMARDs: conventional synthetic disease-modifying antirheumatic drugs; bDMARDs: biological disease-modifying antirheumatic drugs.

**Fig 2:**
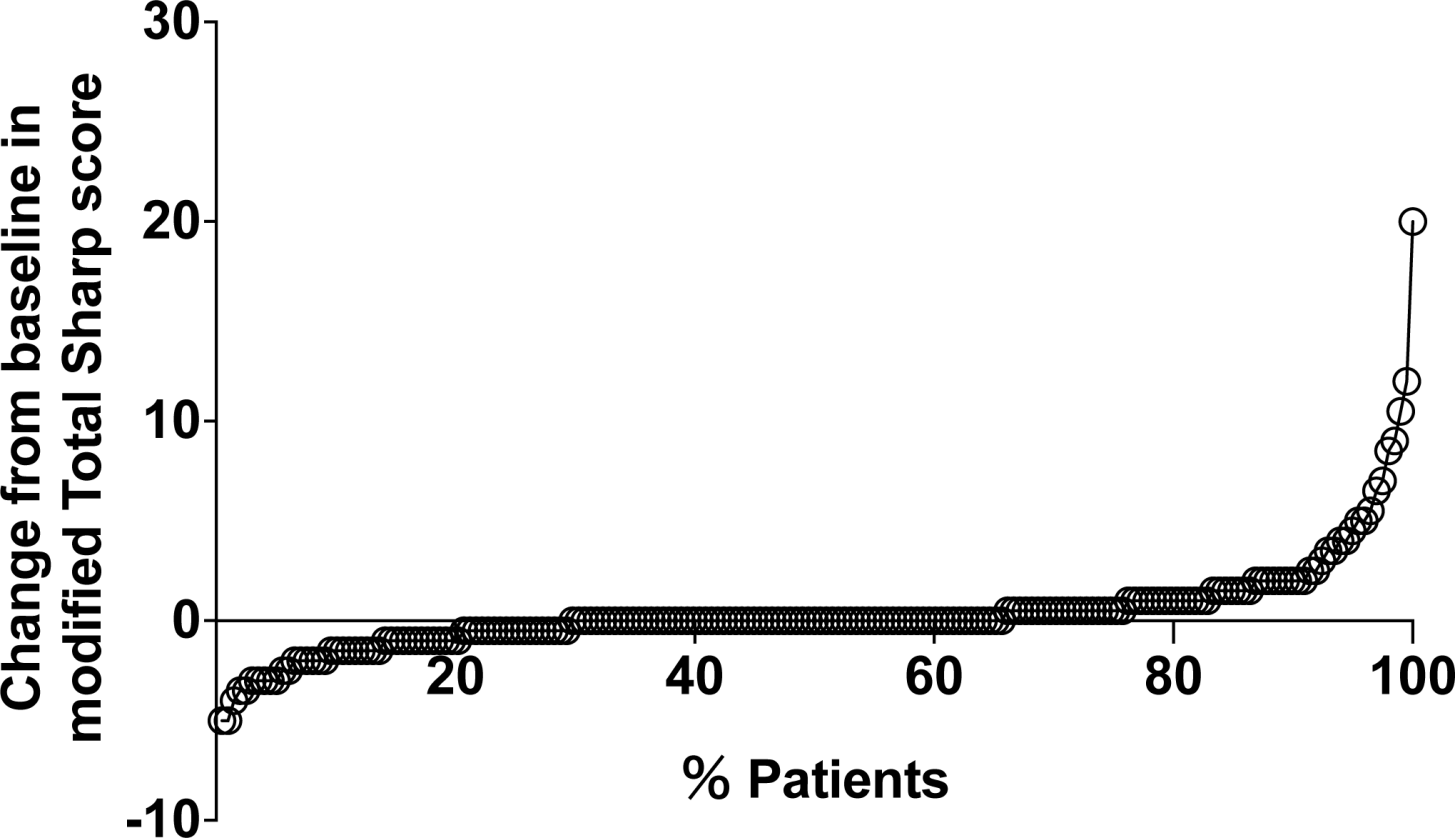
Cumulative probability plots of actual radiographic progression assessed by mTSS (U/year) in the cohort (n = 198). mTSS: modified total Sharp score.

The therapeutic course during the 1 year following the baseline is shown in Fig 1. CRRP was observed in 15 of the 198 patients (7.6%). Among the 198 patients, 142 (72%) achieved sustained clinical remission or LDA during the 1-year observation. Cumulative probability plots during the 1 year post-baseline as assessed by mTSS are shown in Fig 2.

### Prediction of CRRP at 1 year in the RA patients

To determine which variables are associated with the development of CRRP at 1 year, we evaluated the 21 variables shown in Table 1. We found that the following eight variables were significantly associated with CRRP in the univariate analyses: ACPA positivity, time-integrated DAS28-ESR during the 1 year post-baseline, relapse during the follow-up, the CRP level at baseline, the HAQ score at baseline, the total mTSS at baseline, the erosion score at baseline, and the joint space narrowing score at baseline.

**Fig 3:**
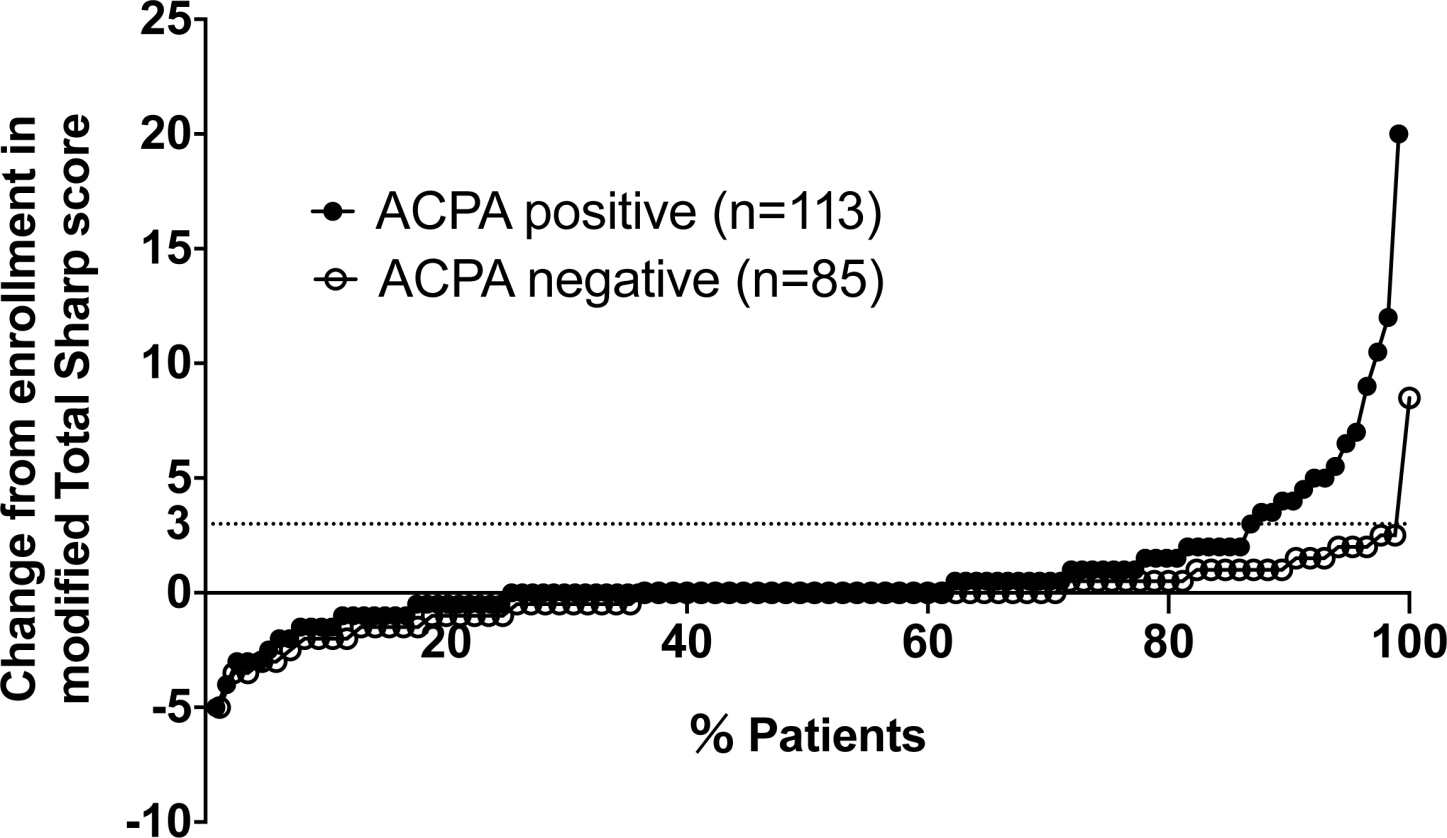
Cumulative probability plots of changes in mTSS for 1 year in the ACPA-positive patients versus the ACPA-negative patients. ACPA: anti-citrullinated peptide antibodies; mTSS: modified total Sharp score.

We selected these variables for a logistic regression analysis and determined the final model by selecting the variables with p-values <0.05 in the first model and identified three independent prognostic factors of CRRP, as follows: ACPA positivity (odds ratio [OR] = 15.2, 95% confidence interval [CI] 2.64–299, p = 0.0007), time-integrated DAS28-ESR during the 1 year post-baseline (7.85-unit increase, OR = 1.83, 95%CI 1.03–3.45, p = 0.036), and mTSS at baseline (13-unit increase, OR = 1.22, 95%CI 1.06–1.42, p = 0.0073) (Table 2). We also show logistic analyses with all variables converted to continuous variables (Table 3) or binary variables (Table 4). These binary variables are determined by constructing ROC curves. Taken together, we determined that ACPA positivity, time-integrated DAS28-ESR during the 1 year post-baseline and mTSS at baseline are independent prognostic factors of CRRP. The cumulative probability plots of changes in mTSS for 1 year in the ACPA-positive patients (n = 113) versus the ACPA-negative patients (n = 85) are shown in Fig 3.

**Table 2:** Prediction of CRRP in multiple logistic regression analysis*.

Variable	Model 1			Final model		
	OR (95%CI)	Unit	p-value	OR (95%CI)	Unit	p-value
ACPA positive	14.02 (2.42–275)		0.0012	15.2 (2.64–299)		0.0007
Time-integrated DAS28-ESR	1.83 (1.03–3.45)	7.85	0.037	1.83 (1.03–3.45)	7.85	0.036
HAQ at baseline	1.09 (0.67–1.68)	0.31	0.69			
mTSS at baseline	1.93 (1.13–2.95)	42.2	0.0133	1.87 (1.18–3.08)	42.2	0.0073
CRP at baseline	1.15 (0.68–1.59)	1	0.51			

*Odds ratio (OR), 95% confidence interval (95%CI), unit change for the continuous explanatory variables (Unit) and p-value in model 1 or final model are shown.

ACPA: anti-citrullinated peptide antibodies; DAS28-ESR: Disease Activity Score 28 joints-erythrocyte sedimentation rate; mTSS: modified total Sharp score; CRP: C-reactive protein.

**Table 3:** Prediction of CRRP in multiple logistic regression analysis (continuous variables)*.

Variables	model 1			final model		
	OR (95%CI)	Unit	p-value	OR (95%CI)	Unit	p-value
ACPA titer	1.76 (1.11–2.74)	96	0.017	1.76 (1.12–2.70)	96	0.017
Time-integrated DAS28-ESR	1.83 (1.03–3.45)	7.85	0.0075	1.83 (1.03–3.45)	7.85	0.0065
HAQ at baseline	1.09 (0.67–1.68)	0.31	0.35			
mTSS at baseline	1.93 (1.13–2.95)	42.2	0.012	1.87 (1.18–3.08)	42.2	0.0061
CRP at baseline	1.15 (0.68–1.59)	1	0.37			

*Odds ratio (OR), 95% confidence interval (95%CI), unit change for the continuous explanatory variables (Unit) and p-value in model 1 or final model are shown.

ACPA: anti-citrullinated peptide antibodies; DAS28-ESR: Disease Activity Score 28 joints-erythrocyte sedimentation rate; mTSS: modified total Sharp score; CRP: C-reactive protein.

**Table 4:** Prediction of CRRP in multiple logistic regression analysis (binary variables)*.

Variables	model 1		final model	
	OR (95%CI)	p-value	OR (95%CI)	p-value
ACPA >48.1 (U/ml)	35.0 (6.11–674)	<0.0001	37.0 (6.64–700)	<0.0001
Time-integrated DAS28-ESR >35.7	4.09 (0.99–17.9)	0.051	4.16 (1.08–16.5)	0.039
HAQ at baseline >0.125	2.25 (0.53–9.63)	0.26		
mTSS at baseline >27	3.84 (1.04–15.5)	0.043	4.62 (1.32–18.1)	0.017
CRP at baseline >0.25 (mg/dl)	1.37 (0.32–5.85)	0.67		

*Odds ratio (OR), 95% confidence interval (95%CI) and p-value in model 1 or final model are shown.

ACPA: anti-citrullinated peptide antibodies; DAS28-ESR: Disease Activity Score 28 joints-erythrocyte sedimentation rate; mTSS: modified total Sharp score; CRP: C-reactive protein. The cut-off values were determined by constructing a receiver operator characteristic (ROC) curve.

Since several studies have demonstrated better radiographic response of bDMARDs above csDMARDs, we performed a sensitivity analysis in which patients treated with bDMARDs are omitted, showing that all of the results did not change significantly after the exclusion (data not shown).

### Prediction of CRRP at 1 year post-baseline in subgroups defined by disease activity

To further examine predictors of radiographic progression in the absence of inflammatory activity for its potential clinical utility, we stratified patients into two groups according to their disease activity at entry: remission (n = 120) or LDA (n = 78). The patients’ characteristics according to disease activity are shown in Table 5. There was no significant difference in the percentage of CRRP between the two groups (remission, 6%; LDA, 10%; p = 0.28).

**Table 5:** Patients’ characteristics with disease activity (univariate analyses)*.

Variables	Remission (n = 120)	LDA (n = 78)	p-value
Demographic:			
Age, yrs	59.9 (12.6)	58.1 (11.8)	0.22
Female, n (%)	85 (71)	59 (76)	0.52
Disease characteristics:			
Disease duration, years	5.2 (2.6–8.8)	4.8 (2.5–7.7)	0.33
RF positive, n (%)	79 (66)	61 (78)	0.079
ACPA positive, n (%)	65 (54)	48 (62)	0.38
RF titer*, U/ml	49 (16–117)	67.7 (26.2–135)	0.14
ACPA titer*, U/ml	67.4 (9.5–105)	70.1 (20.9–100)	0.77
Disease activity:			
DAS28-ESR at baseline	1.95 (0.41)	2.90 (0.18)	<0.001
Time-integrated DAS28-ESR	25.2 (21.3–29.6)	33.3 (28.5–37.1)	<0.001
Relapse during the follow up	26 (22)	30 (38)	0.015
CRP at baseline, mg/dl	0.10 (0.05–0.20)	0.30 (0.15–0.54)	<0.001
ESR at baseline, mm/h	11 (6–15)	15 (11–27)	<0.001
HAQ at baseline	0.12 (0.28)	0.22 (0.35)	0.012
Radiographs:			
mTSS at baseline	11 (7.5–51)	22.5 (5–28)	0.0071
Erosion score at baseline	6 (3–16.5)	12.5 (5–30)	0.0064
JSN score at baseline	4 (1–12)	7 (2–24)	0.012
CRRP, n (%)	7 (6)	8 (10)	0.28
Treatment:			
Methotrexate use, n (%)	93 (78)	63 (81)	0.72
Dose of Methotrexate at baseline, mg/week	6.8 (2.0)	7.0 (2.0)	0.43
Maximum dose of Methotrexate during 1 year, mg/week	7.6 (2.2)	7.9 (1.9)	0.23
Prednisolone use, n (%)	44 (37)	32 (41)	0.55
bDMARDs introduction	4 (3)	4 (5)	0.71
Strengthened csDMARDs, n (%)	35 (29)	24 (31)	0.87

*Mean values (SD), median (interquartile range) or number (percentages) are shown.

P-values were established using Fisher’s exact test or the Mann-Whitney U-test. CRRP: clinically relevant radiographic progression; RF: rheumatoid factor; ACPA: anti-citrullinated peptide antibodies; DAS28-ESR: Disease Activity Score 28 joints-erythrocyte sedimentation rate; CRP: C-reactive protein; HAQ: Health Assessment Questionnaire; mTSS: modified total Sharp score; JSN: joint space narrowing; bDMARD: biological disease-modifying antirheumatic drug.

We subsequently performed a Firth’s bias-reduced penalized-likelihood logistic regression analysis by using the final model created in this study. In the group with remission, ACPA positivity at baseline (OR = 13.3, 95%CI 1.46–176, p = 0.017), and mTSS at baseline (11-unit increase, OR = 1.27, 95%CI 1.02–1.64, p = 0.0316) are independent variables to predict the development of CRRP. Especially, all of the patients who developed CRRP had positive ACPA at baseline. Meanwhile, we found that ACPA positivity at baseline (OR = 8.10, 95%CI 1.14–181, p = 0.0345) was the only variable for predicting CRRP in the group with LDA at enrollment. Taken together, our results show that the prognostic factors of CRRP differ according to the disease activity.

## Discussion

Although achieving clinical remission is a primary goal of RA, it should be noted that some RA patients in remission experience joint destruction. In the present study we sought to determine the predictive factors for CRRP in bDMARD-naïve RA patients who achieved remission or LDA by treatment with conventional DMARDs, and our analyses revealed that ACPA positivity was the strongest predictor of CRRP among these patients.

Studies that investigated early RA patients with high disease activity revealed that autoantibodies such as ACPA and rheumatoid factor (RF) were predictive factors for CRRP [7, 8, 24]. However, little is known the effect of autoantibodies on CRRP in patients with RA who achieve remission or LDA. Our present findings demonstrated a direct association between ACPA positivity and CRRP among Japanese RA patients in real-world daily practices, which suggests that physicians should consider the possibility of subclinical or residual synovitis in RA patients with ACPA positivity even if the patients have achieved remisson by csDMARD treatment.

Our subgroup analysis showed that ACPA positivity is a potent prognostic factor toward CRRP among patients with remission compared with those with LDA. Of note, a systematic review and meta-analysis reported that the presence of synovial hypertrophy on US, power Doppler activity or synovitis shown by MRI at baseline in RA patients in clinical remission were each significantly associated with structural progression at 1 year, even in asymptomatic joints [25]. Thus, it is important for clinicians to assess subclinical/residual synovitis by using MRI or US in ACPA-positive patients who achived remission.

Schett et al. recently reviewed the predictors of relapse in RA patients in clinical remission during the tapering of biologic and conventional DMARDs [26]. With respect to serum biomarkers, the best-studied predictor of relapse to date is ACPA positivity, and the second is RF positivity [26]. The clinical situations are different between this review and our cohort since we did not intend to taper or stop the DMARDs; however, ACPA is thought to be an indispensable biomarker of unfavorable outcome regardless of continuing, tapering or stopping DMARDs in RA patients with clinical remission or LDA. In fact, in the present study the rate of relapse during the 1-year observation was 35% in the ACPA-positive patients and 19% in the ACPA-negative patients (p = 0.011).

In the present study RF, unlike ACPA, did not predict the development of CRRP. It is well known that RF positivity, unlike that of ACPA, can be changed to negativity by DMARD treatment [27, 28]. The ACPA and RF serostatus in our patients was obtained at the latest data collection, and thus the serostatus of RF, but not that of ACPA, might have been influenced by the introduction of DMARDs and might not accurately reflect the immunological activities of the patients.

The multiple logistic regression analysis revealed that time-integrated DAS28-ESR during the 1 year post-baseline was also significantly associated with CRRP. This finding is consistent with the previous report that radiographic progression was prominent in the subgroup of patients with disease exacerbation in a cohort of European RA patients in clinical remission at enrollment [9]. Our data also confirm the importance of the maintenance of favorable clinical disease activity achieved by the T2T strategy in the EULAR recommendations [3, 29].

Our group [16] and others [24, 30] reported that the CRP level at baseline is a predictive factor for CRRP in RA patients with moderate to high disease activity. In contrast, the present investigation of RA patients in remission or with LDA showed that the CRP level at baseline was not associated with CRRP. Taken together, these findings indicated that the CRP level at baseline is a predictive factor for CRRP only in RA patients with obvious disease activity, since the patients' CRP levels at enrollment were considerably low (median 0.15 mg/dl). In addition, joint damage at baseline was reported to be a significant predictor of CRRP in RA patients with high disease activity [7, 8]. In line with these observations, the multiple logistic regression analysis in the present study revealed that the mTSS at baseline was an independent variable to predict CRRP; this finding reconfirms that joint damage sustains joint inflammation [31]. These processes might be more apparent in RA patients in remission or with LDA compared to patients with moderate to high disease activity, because our present findings demonstrated that mTSS but not CRP at baseline is predictive of CRRP.

There are some study limitations to acknowledge. First, the follow-up period was only 1 year. Since the structural damage in RA progresses over several years, long-term verification studies are needed to confirm our results. Second, although Takahashi et al. reported that Japanese patients with RA had higher concentrations of the active form of methotrexate (MTX)-polyglutamate in their red blood cells than RA patients in other counties [32], the dosage of MTX in our cohort was lower than that in the Takahashi cohort. Accordingly, we cannot exclude the possibility that the efficacy of MTX was underestimated in the present study. Finally, we were unable to investigate the period during which our patients achieved remission or LDA prior to their entry in the study. Recruited patients were in remission at the time of this entry, but we did not have detailed information on the recent treatment change and the clinical course before the start of observation. There is a possibility that strengthening treatment just before starting observation have influenced the outcome of this study.

In conclusion, our findings indicate that CRRP in patients with rheumatoid arthritis in remission or LDA was associated with ACPA positivity, mTSS at baseline and time-integrated DAS28-ESR, the strongest predictor being ACPA. Since structural damage is strongly associated with functional impairment in RA patients [33], our results offer important information that can be used to help avoid radiographic progression in RA patients who have achieved a favorable clinical response with DMARD treatment.

## Abbreviations

ABT: abatacepet
ACPA: anti-citrullinated peptide antibodies
ADA: adalimumab
bDMARD: biological disease-modifying antirheumatic drug
CI: confidence interval
CRP: C-reactive protein
CRRP: clinically relevant radiographic progression
csDMARD: conventional synthetic disease-modifying antirheumatic drug
DAS28: Disease Activity Score in 28 joints
ESR: erythrocyte sedimentation rate
ETN: etanercept
EULAR: European League Against Rheumatism
HAQ: Health Assessment Questionnaire
IFX: infliximab
LDA: low disease activity
MRI: Magnetic resonance imaging
mTSS: modified total Sharp score
MTX: methotrexate
OR: odds ratio
RA: rheumatoid arthritis
RF: rheumatoid factor
T2T: treat-to-target
TCZ: tocilizumab
US: ultrasonography
